# Health system delay and risk factors in pulmonary tuberculosis diagnosis before and during the COVID-19 epidemic: a multi-center survey in China

**DOI:** 10.3389/fpubh.2025.1526774

**Published:** 2025-02-26

**Authors:** Mingkuan Fan, Yushu Liu, Kui Liu, Xiaoqiu Liu, Yuhong Li, Tao Li, Canyou Zhang, Hui Zhang, Jun Cheng

**Affiliations:** ^1^National Center for Tuberculosis Control and Prevention, Chinese Center for Disease Control and Prevention, Beijing, China; ^2^Medical College of Xiangyang Polytechnic, Xiangyang, Hubei, China; ^3^Department of Tuberculosis Control and Prevention, Zhejiang Provincial Center for Disease Control and Prevention, Hangzhou, Zhejiang, China; ^4^National Key Laboratory of Intelligent Tracking and Forecasting for Infectious Diseases, Chinese Center for Disease Control and Prevention, Beijing, China

**Keywords:** tuberculosis, lung, delay, diagnosis, risk factors

## Abstract

**Background:**

Understanding health system delay (HSD) in pulmonary tuberculosis (PTB) diagnosis aids in tailoring interventions for case detection and curbing transmission. However, recent nationwide studies on HSD in PTB diagnosis have been scarce. This study assesses HSD and its risk factors in China, taking into account the impact of the COVID-19 epidemic.

**Methods:**

Patients diagnosed with PTB between 2019 and 2022 were selected using a multistage stratified clustering method. A semi-structured questionnaire was employed to assess HSD, which was defined as the interval between the patient’s initial visit to a health facility and the definitive PTB diagnosis. The HSD was then compared between 2019 (before the epidemic) and 2020–2022 (during the epidemic). Factors associated with long health system delay (LHSD, defined as HSD > 14 days) were examined using both univariate and multivariate analyses with chi-square tests and binary logistic regression, respectively.

**Results:**

In total, 958 patients with PTB were analyzed: 478 before and 480 during the epidemic. The HSD was 14 (interquartile range, 7–30) days for all patients, and the HSD before and during the epidemic also shared this value. A total of 199 patients (20.8%) had LHSD. LHSD was more prevalent in patients presenting solely with cough and expectoration (Odds ratio [OR]: 1.482, 95% confidence interval [CI]: 1.015–2.162) and those visiting ≥2 health facilities before definitive diagnosis (2 health facilities: OR = 2.469, 95%CI: 1.239–4.920; ≥3 health facilities: OR = 8.306, 95%CI: 4.032–17.111). Additionally, patients with negative bacteriological results were independently associated with higher LHSD risk (OR = 1.485, 95%CI: 1.060–2.080).

**Conclusion:**

In China, HSD in PTB diagnosis remains relatively low and is primarily mediated by factors associated with health providers. No significant impact on HSD from the COVID-19 epidemic has been found. Implementing targeted training programs to enhance health providers’ awareness of chronic respiratory symptoms and maintain vigilance for PTB; strengthening presumptive PTB identification capabilities at grassroots health facilities, and promoting the use of *Mycobacterium tuberculosis* (MTB) bacteriological technologies are recommended to shorten the HSD.

## 1 Introduction

Tuberculosis (TB), caused by *Mycobacterium tuberculosis* (MTB), remains a major global public health challenge as one of the top infectious disease killers worldwide (1). According to the latest World Health Organization (WHO) estimates, TB was responsible for approximately 10.8 million new cases and 1.25 million deaths globally in 2023 (1). In response to this persistent epidemic, the WHO launched the “End TB Strategy” with ambitious targets for 2035, including a 90% reduction in TB incidence and 95% reduction in TB deaths compared to 2015 levels (2). Early case detection has been identified as a fundamental component of this strategy, as prompt TB diagnosis and subsequent correct treatment can alleviate patients’ suffering and lead to positive prognosis (3). More importantly, it can reduce the duration of MTB transmission in the community. Therefore, minimizing diagnostic delays is crucial for achieving effective TB control and meeting global elimination targets.

According to the WHO (4), diagnostic delay can be divided into two segments, the former is the interval from the onset of symptoms to the first visit to a health provider, which is called patient delay, and the interval between the initial health facility visit and definitive TB diagnosis, also known as health system delay (HSD). Extensive research conducted in this field has revealed considerable spatiotemporal heterogeneity in the degree and determinants of HSD (5). The median HSD across different countries spans from 2 to 128.5 days in low- and middle- income countries in a systematic review study (6). Chakma et al. (7) reported that the HSD among TB patients in Portugal exhibited a slight increasing trend over time from 2007 to 2018. Regarding the factors influencing HSD, previous studies have demonstrated these factors originate from individuals’ and health providers’ aspects (8, 9). However, significant variations exist across studies regarding the relative importance of these factors (9). This heterogeneity underscores the need for updated, context-specific analyses of HSD patterns and determinants to inform targeted interventions for optimizing TB diagnostic pathways.

China ranks third among 30 countries with the highest burden of new TB cases (1), with pulmonary tuberculosis (PTB) accounting for the majority of the cases (10), making PTB a top priority in infectious disease prevention and control efforts (11). In recent years, several studies have focused on HSD and related factors. Li et al. (12) reported that the average HSD of 50,606 patients with PTB diagnosed from 2016 to 2022 in Gansu province was 6.88 days, with 91.12% of patients being diagnosed within 14 days. A study in Shanghai on patients with PTB diagnosed during 2018–2020 found that, 22.12% of migrants and 16.52% of locals patients experienced HSD exceeding 14 days; and migrant patients and initial care seeking at general hospitals were associated with a higher probability of HSD > 14 days (13). Hao et al. (14) conducted a survey in 2020 and found that the median HSD for patients with PTB from western China was 12 days. Furthermore, the use of molecular biological methods significantly shortened the HSD. However, most of these studies were regional or based on tuberculosis surveillance data, making it difficult to quantify the overall national situation, and surveillance data often lack variables such as number of health facilities patient visited before definitive diagnosis may be related to HSD (15).

Besides, during the COVID-19 epidemic (hereafter referred to as the epidemic), TB services experienced varying degrees of disruption both domestically and internationally, significantly affecting PTB diagnosis and management (16, 17). In response, Chinese health authorities implemented strategic directives to maintain essential TB services while addressing COVID-19 priorities, ensuring the continuity of basic TB diagnosis and treatment (18). The epidemic intensity exhibited substantial fluctuations between January 2020 and November 2022, prompting corresponding adjustments in non-pharmacological interventions (NPIs) (19). Understanding HSD in PTB diagnosis within this complex context is essential for developing resilient TB diagnostic services in the future. However, existing research in China has primarily focused on localized regions and the initial epidemic phases (20, 21), leaving the nationwide impact on HSD largely unexplored. To address this knowledge gap, our study employs comprehensive patient surveys, incorporating COVID-19-related factors, to evaluate the current status and identify determinants of HSD in PTB diagnosis across China. The findings will provide critical evidence to inform policy development and strengthen TB control strategies in the post-epidemic era.

## 2 Methods

### 2.1 Study design and settings

This is a retrospective survey to investigate the HSD in PTB diagnosis. The study sites were chosen by multistage random sampling method. Taking into account the evolving COVID-19 control policies in China (22), we divided the epidemic period into four distinct stages: stage I, January to April 2020; stage II, May 2020 to July 2021; stage III, August 2021 to March 2022; and stage IV, April 2022 to November 2022. Meanwhile, we classified all counties into three levels based on the stringency of NPIs implemented for epidemic during this period, namely strict, moderate, and general. The classification of NPI stringency was determined by experts from the China CDC. Here are the methodologies employed for selecting the study sites during stage I.

Initially, we picked a month from January to April 2020 to serve as the starting point for selecting participants during the epidemic, with this month meet the following conditions: (1) at least two provinces reported more than one hundred COVID-19 cases. Each of these provinces had at least one county implementing strict NPIs, one implementing moderate NPIs, and one implementing general NPIs, all lasting for more than 15 days during that month. (2) Each of the selected counties reported no fewer than 120 PTB cases in 2019. If more than 1 month meet these conditions, using the random number method to select one. Next, selected two provinces after determining the month, if more than two provinces meet the requirements, randomly select two of them. Then, in the selected provinces, randomly chose one county each that implemented strict, moderate, and general NPIs during the selected month. Finally, for stage I, six counties located in Hubei and Henan provinces were selected as study sites, and the selection of patients began during the epidemic, specifically in March 2020.

**Figure 1:**
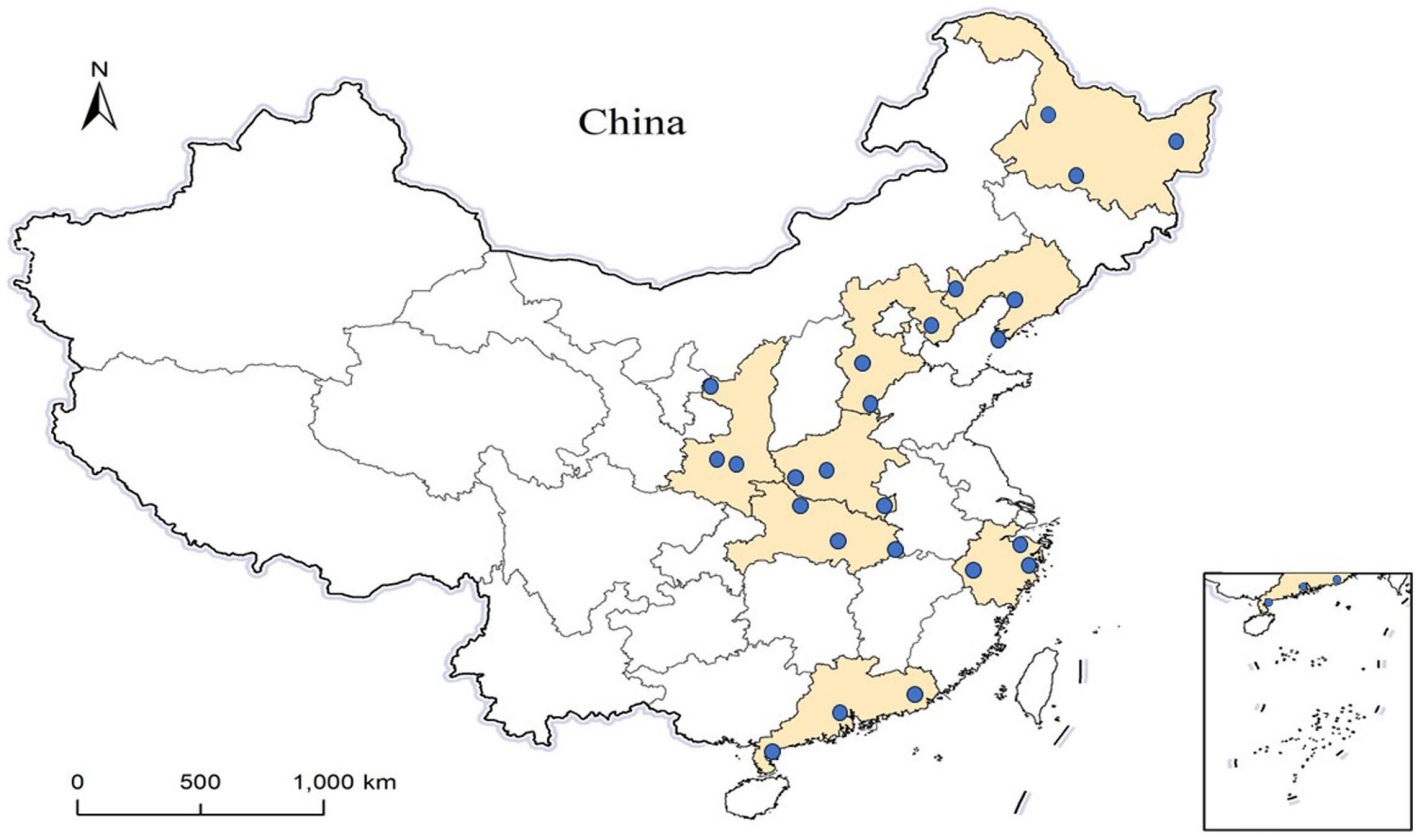
The location of the 24 study sites. The colored areas are 8 provinces and the blue dots represent the geographical locations of the selected counties.

For the other three stages, the methodology for study sites selection was similar to that of stage I, ensuring that no province was chosen more than once. Eventually, 24 counties across 8 provinces were selected (Figure 1). The starting time patients to be selected during the epidemic began in stage II-IV were January 2021, November 2021, and May 2022, respectively.

### 2.2 Sample size

The sample size was estimated for this study using the formula, *n* = z^2^*p*(1-*p*)/*δ*^2^. Taking into account a 95% confidence level, a proportion of LHSD within PTB patients (*p* = 0.36) derived from a meta-analysis conducted in China (23), a 5% allowable error, and a design effect of 2.5 due to the cluster sampling method (24), the calculation was performed. Thus, at least 885 PTB participants need to be included in the study. After dividing by 24, approximately 37 patients were needed in each county. Therefore, we decided to select 40 patients in each county, with 20 before and 20 during the epidemic.

### 2.3 Participants

Using a multistage clustering sampling method to select participants in the TB-designated hospitals of the selected counties, patients diagnosed with PTB and notified to the Chinese Tuberculosis Information Management System (TBIMS) were selected. Notably, in China, it is a legal requirement for all confirmed PTB cases to be registered within TBIMS (25).

For the six counties selected in stage I, starting from the first patient registered in March 2020, a consecutive series of 20 patients were contacted. In case of death, loss to follow-up, or refusal to participate, these patients were replaced sequentially based on their registration numbers. Using the same method, 20 patients from each of 6 counties in 2019 were selected, consistent with the epidemic period starting March 2019. For the remaining 18 counties in stages II to IV, the selection of patients began during the epidemic at determined month, and patients before the epidemic were selected starting from the corresponding month in 2019. The patient selection methodology in these counties remained consistent with that used for the 6 counties in stage I.

### 2.4 Data collection

Data were collected using a pre-tested, interviewer-administered semi-structured questionnaire between September and December 2023. The survey covered the participants’ sociodemographic characteristics (including sex, age, education level, occupation, marital status, residence), TB treatment history, PTB symptoms, pathway, and duration (days) from their first health facility visit to definitive diagnosis. The case-finding method, health facility types, and number of health facilities visited before diagnosis were determined based on the patients’ pathways. Specifically, the patient’s diagnosis after self-presentation to a health facility due to PTB symptoms was classified as passive case-finding, whereas other scenarios were classified as active case-finding. Health facilities were classified into four types: grassroots health facilities (including village clinics, private clinics, and township health centers), general hospitals, other non-TB-designated hospitals, and TB-designated hospitals. Repeated visits to the same health facility were counted as a single facility. Additionally, data on TB bacteriological results were collected through a review of medical records. For patients with communication barriers, under 15 years of old, or with mental disorders, a caregiver or guardian was asked to respond on their behalf.

### 2.5 Relevant definitions

Based on previous study (4), HSD was defined as the time interval from the patient’s initial visit to a health facility before the definitive diagnosis of PTB. Long health system delay (LHSD) occurs when this interval exceeds 14 days (26).

PTB symptoms: at least one of the following symptoms persisting or recurring prior to PTB diagnosis: cough and expectoration, hemoptysis, fever, chest pain, night sweats, fatigue, appetite loss, or weight loss.

Bacteriological results: results of the MTB test when patients were diagnosed by sputum smear microscopy, culture, or PCR; a “positive” result indicates the detection of MTB, while a “negative” result indicates that MTB was not detected.

### 2.6 Ethical approval

Ethical approval was obtained from the Ethics Committee of the Chinese Center for Disease Control and Prevention. All the study participants provided informed consent. The data were anonymized before further analyses.

### 2.7 Statistical analysis

Survey data were subjected to a double-entry process using Epidata3.1 (The Epidata Association Odense, Denmark) to ensure precision before being exported to a Microsoft Excel (version 2016; Microsoft Corporation, WA, United States) spreadsheet. Statistical analyses were performed using SPSS22.0™ (IBM Corp. Released 2013. IBM SPSS Statistics for Windows, Version 22.0. Armonk, NY: IBM Corp.). Diagrams were created using R software (version 4.4.1, Auckland, New Zealand) and Sankey MATIC (an online Sankey diagram tool).

The statistical analysis was conducted using appropriate methods based on variable types and distribution characteristics. Categorical variables were presented as frequencies and percentages, with between-group comparisons performed using chi-square tests. For continuous variables, we first assessed data distribution using the one-sample Kolmogorov–Smirnov test (K–S test). Normally distributed data were expressed as mean ± standard deviation (SD), while non-normally distributed variables were summarized using median and interquartile range (IQR). To compare HSD distributions between before and during the epidemic, we employed the two-sample K–S test. Group comparisons of HSD duration were conducted using either the Student’s *t*-test (for normally distributed data) or the Mann–Whitney *U* test (for non-normal distributions), as appropriate. Univariate analysis was performed using chi-square tests to identify potential predictors of LHSD. To minimize the exclusion of potentially significant variables, we adopted a liberal *p*-value threshold of ≤0.2 for inclusion in subsequent multivariate analysis. Significant variables from the univariate analysis were then included in a binary logistic regression model to identify independent determinants of LHSD. The final multivariate model results were expressed as adjusted odds ratios (OR) with corresponding 95% confidence intervals (CI). A two-tailed *p*-value<0.05 was considered statistically significant.

## 3 Results

### 3.1 Characteristics of participants

**Figure 2:**
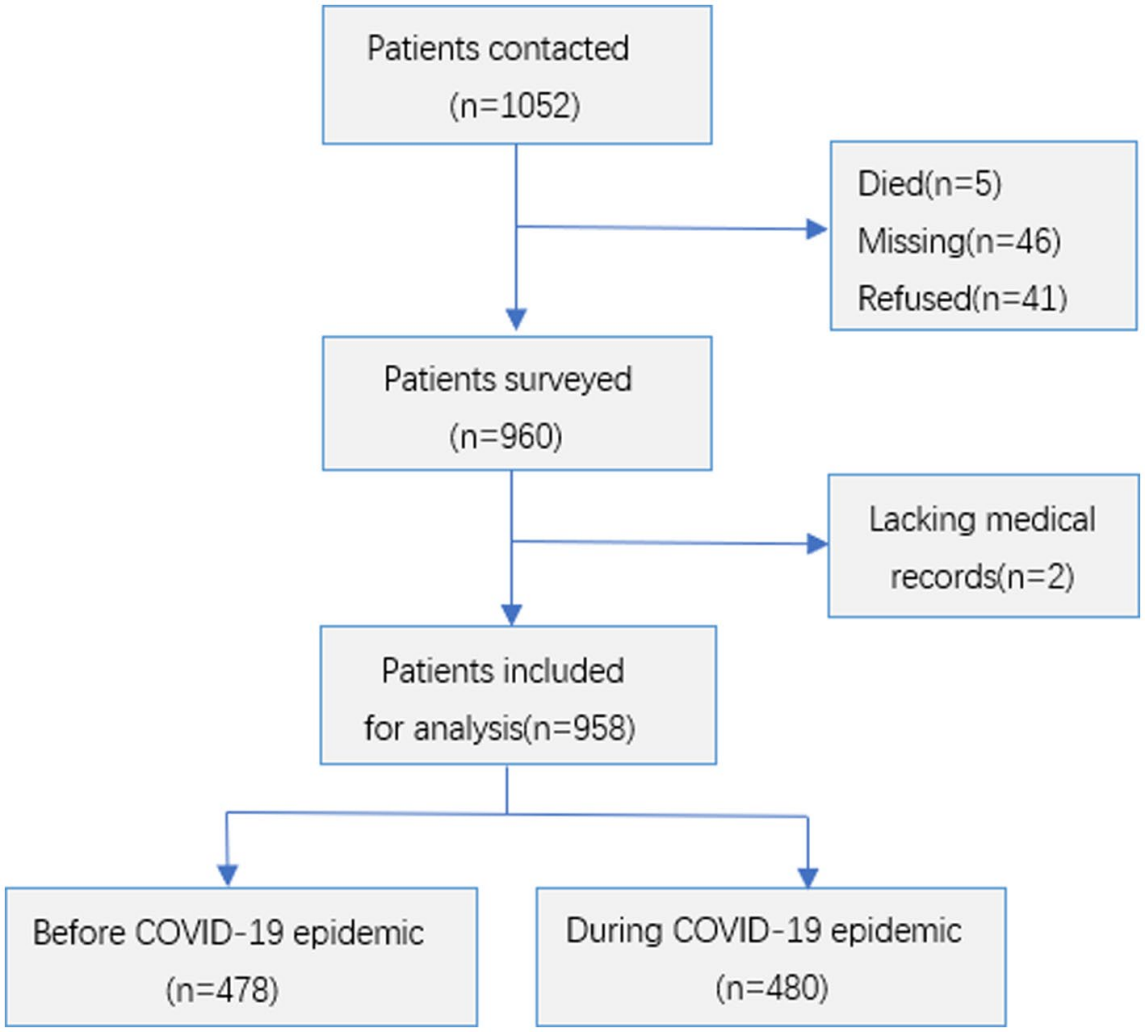
Flow diagram of PTB cases including for health system delay analysis.

A total of 1,052 PTB cases were initially contacted for participation. After excluding 92 cases due to death (*n* = 5), loss to follow-up (*n* = 46), and refusal to participate (*n* = 41), 960 patients completed the survey. Two additional cases were excluded due to missing medical records, resulting in 958 cases included in the final analysis. The cohort comprised 478 patients diagnosed before the epidemic and 480 patients diagnosed during the epidemic period (Figure 2). Comparative analysis revealed no significant differences between analyzed and excluded patients in terms of: mean age (48.4 ± 17.5 years vs. 48.8 ± 16.7 years; *p* = 0.846), gender distribution (68.6% vs. 73.4% male; *p* = 0.334).

The majority of participants were male 657 (68.6%). Slightly more than half of the patients were aged between 35 and 64. Nearly two-thirds of the participants had completed junior middle school or below. One-third of participants were engaged in agricultural labor. Nearly 74% of patients were married or cohabiting. Of all the participants, 563 (58.8%) lived in urban areas. A total of 72 (7.5%) had a history of TB treatment. Up to 759 (79.2%) patients presented with PTB symptoms, and 250 (26.1%) only had cough and expectoration. Among them, 648 patients (67.6%) visited two health facilities before being confirmed. There were no statistical differences in the distribution of the above characteristics between patients diagnosed before and during the epidemic. Of the 958 patients, 459 (47.9%) were confirmed as bacteriologically positive. Among the 958 patients included in the analysis, 459 (47.9%) were bacteriologically confirmed through positive test results. Active case-finding accounted for 190 (19.8%) of all diagnoses. Comparative analysis revealed significant differences between patients diagnosed before and during the epidemic: Higher proportion of bacteriologically positive cases during the epidemic (*p* < 0.05) and increased rate of diagnosis through active case-finding during the epidemic (*p*<0.05). More details were displayed in Table 1.

**Table 1:** Characteristics of the participants included for health system delay analysis.

Characteristics	Total (*n* = 958)	Pre-epidemic (*n* = 478)	During epidemic (*n* = 480)	*p*-value
Sex, *n* (%)				0.560
Male	657(68.6)	332(69.5)	325(67.7)	
Female	301(31.4)	146(30.5)	155(32.3)	
Age (year), mean ± SD	48.4 ± 17.5	48.3 ± 17.0	48.5 ± 17.0	0.824
Education level, *n* (%)				0.097
Junior middle school or below	633(66.1)	328(68.6)	305(63.5)	
Above junior middle school	325(33.9)	150(31.4)	175(36.5)	
Occupation, *n* (%)				0.668
Agricultural workers	324(33.8)	168(35.1)	156(32.5)	
Industrial workers	168(17.5)	81(16.9)	87(18.1)	
Cadre and staff^a^	69(7.2)	35(7.3)	34(7.1)	
Students	60(6.3)	30(6.3)	30(6.3)	
Retiree	58(6.1)	28(5.9)	30(6.3)	
Houseworkers and un-employed	174(18.2)	92(19.2)	82(17.1)	
Others	105(11.0)	44(9.2)	61(12.7)	
Marital status, *n* (%)				0.224
Unmarried	170(17.7)	93(19.5)	77(16.0)	
Married or cohabitate	707(73.8)	341(71.3)	366(76.3)	
Divorce or widow	81(8.5)	44(9.2)	37(7.7)	
Residence, *n* (%)				0.242
Urban	563(58.8)	272(56.9)	291(60.6)	
Rural	395(41.2)	206(43.1)	189(39.4)	
TB treatment history, *n* (%)				0.821
Yes	72(7.5)	35(7.3)	37(7.7)	
No	886(92.5)	443(92.7)	443(92.3)	
Symptoms, *n* (%)				0.147
Others^b^	509(53.1)	262(54.8)	247(51.5)	
Only cough and expectoration	250(26.1)	129(27.0)	121(25.2)	
Asymptomatic	199(20.8)	87(18.2)	112(23.3)	
Number of facilities visited before definitive diagnosis, *n* (%)				0.523
1	114(11.9)	61(12.8)	53(11.0)	
2	648(67.6)	325(68.0)	323(67.3)	
≥3	196(20.5)	92(19.2)	104(21.7)	
Bacteriological results, *n* (%)				0.001
Positive	459(47.9)	204(42.7)	255(53.1)	
Negative	499(52.1)	274(57.3)	225(46.9)	
Case-finding, *n* (%)				0.016
Active	190(19.8)	80(16.7)	110(22.9)	
Passive	768(80.2)	398(83.3)	370(77.1)	

^aIncluding teachers, medical staff, cadres and other brain-based laborers. bPatients with PTB symptoms except the ones with cough and expectoration only.^

### 3.2 Patient pathway

**Figure 3:**
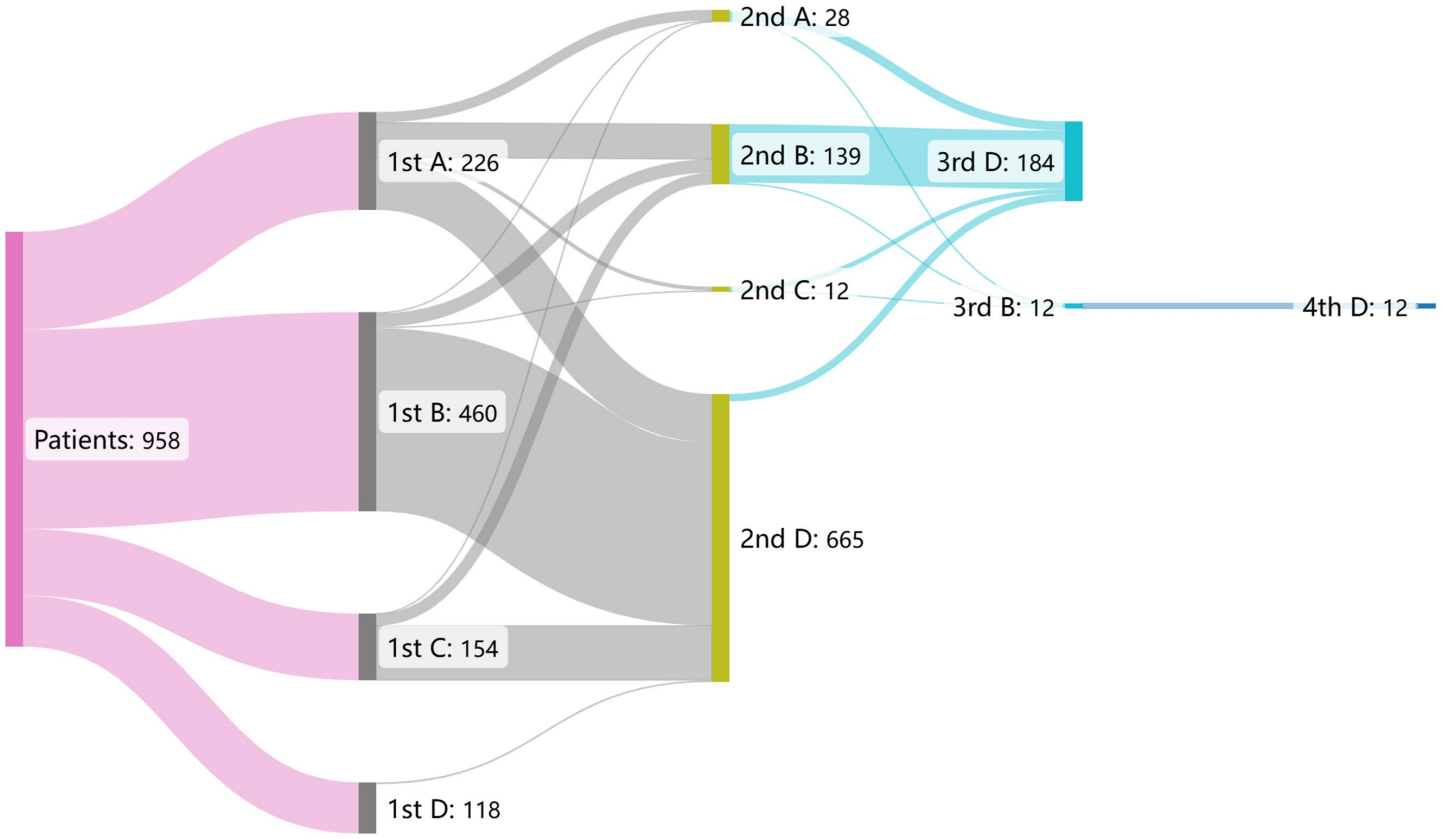
The flow gram of patient pathway before pulmonary tuberculosis diagnosis in 958 patients. The alphabet A–D in the figure represents distinct health facilities. A: grassroots health facilities, B: general hospitals, C: other non-TB-designated hospitals, D: TB-designated hospitals.

All 958 patients received definitive diagnoses at TB-designated hospitals. A total of 114, 648, 184, and 12 patients (11.9, 67.6, 19.2, and 1.3%, respectively) visited 1, 2, 3, and 4 health facilities, respectively, until the definitive diagnosis. Of all patients, 226 (23.6%), 460 (48.0%), 154 (16.1%) initially visited grassroots health facilities, general hospitals, and other non-TB-designated hospitals, respectively; while 118 (12.3%) patients selected TB-designated hospitals as their first-contact health facilities (Figure 3).

Among the 226 patients who chose grassroots health facilities for their first visit, 112 (49.6%) chose TB-designated hospitals as their secondary health provider. This number (proportion) was 423 (92.0%) and 126 (79.7%) for those who initially visited general hospitals and other non-TB-designated hospitals, respectively. Among the 118 patients who first chose TB-designated hospitals, 114 (96.6%) were diagnosed there.

### 3.3 Health system delay

**Figure 4:**
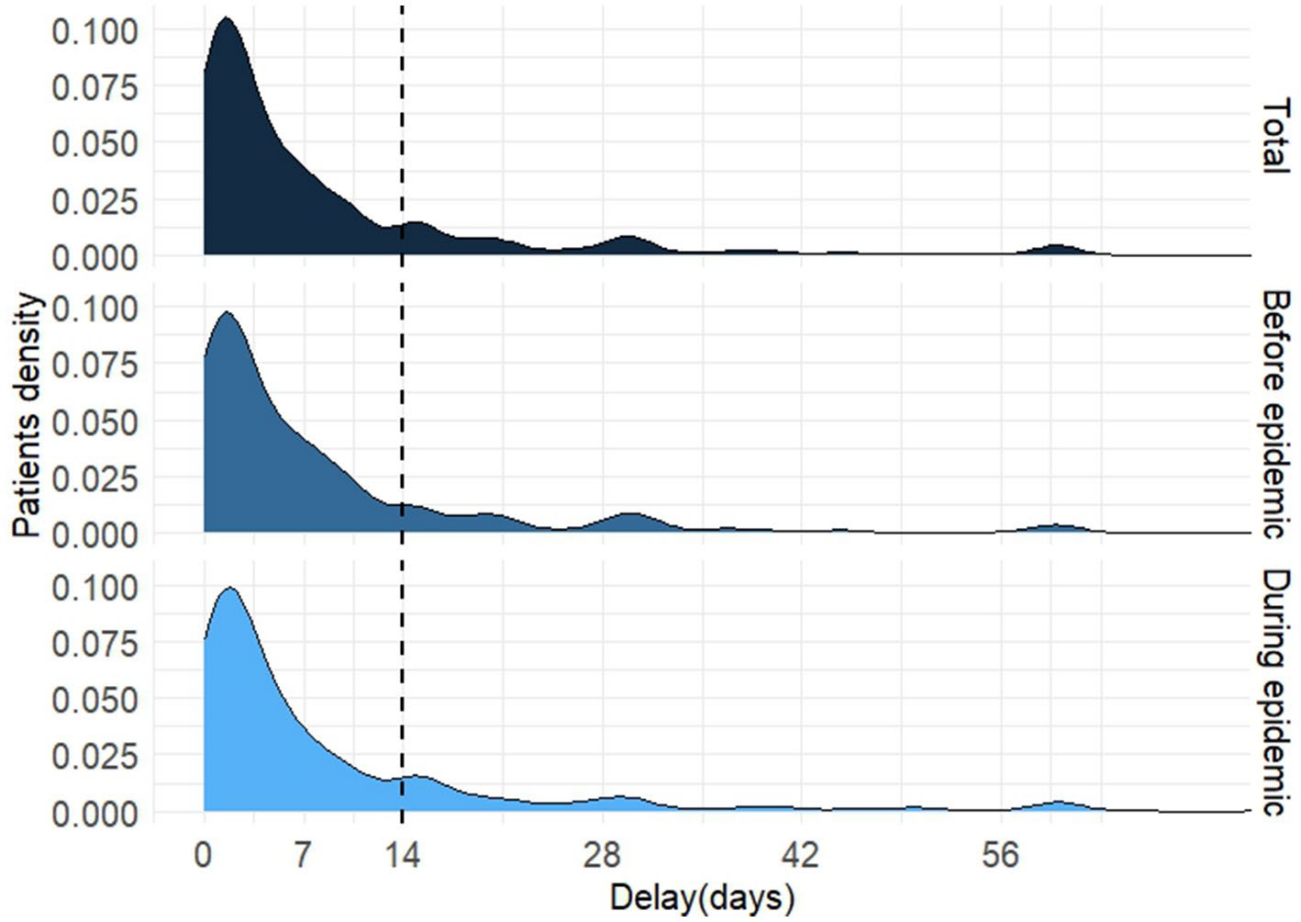
The distribution of health system delay of diagnosis in patients with pulmonary tuberculosis.

Among the 958 patients with PTB, the distribution of HSD was positively skewed, ranging from 0 to 64 days (Figure 4). The K–S test showed no statistical difference in the distribution of HSD before and during the epidemic (*D* = 0.405, *p* = 0.997). The HSD for all participants was 4 (1–10) days, whereas patients diagnosed before and during the epidemic shared the same HSD (4[1–10] days) with no statistical significance (Mann–Whitney *U* test, *p* = 0.793). All the medians of HSD in subcategories varied from 1 to 5 days except for patients visiting ≥3 health facilities before PTB confirmation, who had a much longer HSD (10[4–27] days). Patients in all subcategories, whether diagnosed during or before the epidemic, showed no significant differences in HSD (Table 2).

**Table 2:** Health system delay between patients with pulmonary tuberculosis in different categories.

Categories	Total (*n* = 958)	Pre-epidemic (*n* = 478)	During epidemic (*n* = 480)	*P*-value
Sex				
Male	4(1–10)	4(1–10)	3(1–10)	0.939
Female	4(1–10)	4(1–10)	4(2–10)	0.929
Age (year)				
0–34	3(1–9)	3(1–10)	3(1–7)	0.776
35–64	4(1–10)	3(1–10)	4(1–12)	0.339
65–	5(2–15)	5(2–15)	4(2–11)	0.513
Education level				
Junior middle school or below	4(1–10)	4(1–10)	4(1–12)	0.945
Above junior middle school	3(1–10)	3(1–9)	3(1.5–10)	0.599
Occupation				
Agricultural workers	5(2–13)	6(2–15)	4(2–12)	0.210
Industrial workers	3(1–10)	2(1–9)	3(1–12.5)	0.220
Cadre and staff^a^	3(1–10)	2(1–9.5)	3(2–10)	0.530
Students	3(1–7)	2(1–9)	3(2–7)	0.325
Retiree	4.5(2–10)	5(2–10)	3.5(1–10)	0.458
Houseworkers and un-employed	4(1–10)	4(1.5–8.5)	5(1–15)	0.411
Others	3(1–10)	3(1.5–7)	3(1–13)	0.901
Marital status				
Unmarried	3(1–9)	3(1–10)	3(1–7)	0.922
Married or cohabitate	4(1–10)	4(1–10)	4(1–12)	0.501
Divorce or widow	5(1–15)	6(1.5–19.5)	4(1–10)	0.247
Residence				
Urban	4(1–10)	4(1–9)	4(2–10)	0.187
Rural	3(1–12.5)	4(1–15)	3(1–10)	0.338
TB treatment history				
Yes	2(1–7)	2(1–7)	3(2–10)	0.486
No	4(1–10)	4(1–10)	4(1–10)	0.934
Symptoms				
Others^b^	4(2–10)	5(2–10)	4(2–12)	0.603
Only cough and expectoration	5(2–15)	4(2–10)	5(2–16)	0.434
Asymptomatic	2(1–7)	2(1–7)	3(1–7)	0.169
Number of facilities visited before definitive diagnosis				
1	2(0–5)	2(0–5)	2(1–5)	0.604
2	3(1–10)	3(1–9)	3(1–10)	0.570
≥3	10(4–27)	10(4–22)	10(4–28.5)	0.633
Bacteriological results				
Positive	3(1.5–9)	3(1.5–7)	3(1.5–10)	0.714
Negative	4(1–12)	4.5(1–12)	4(1–12)	0.917
Case-finding				
Active	2(1–7)	2(1–6)	3(1–7)	0.148
Passive	5(2–11.5)	5(2–10)	5(2–12)	0.912

The data in the middle three columns are presented as medians (IQR). ^a^Including teachers, medical staff, cadres and other brain-based laborers. ^b^Patients with PTB symptoms except the ones with cough and expectoration only.

Among all the participants, 13 patients diagnosed during the epidemic recalled that, due to fever symptoms, they were requested to undergo chest CT scans to rule out COVID-19 with prompt detection of PTB. However, another feverish patient tested negative for SARS-CoV-2 nucleic acid test and was considered with cold by doctor, due to the worsening of the illness, a chest CT scan confirmed the diagnosis of PTB 20 days later.

### 3.4 Long health system delay and related factors

A total of 199 (20.8%) participants had experienced LHSD. Univariate analysis revealed no significant association between the LHSD and sex, education level, TB treatment history, or diagnosis period. Similarly, age, occupation, residence, and bacteriological results were not found to be associated with LHSD, with *p*-values slightly exceeding the threshold of 0.05. Marital status, symptoms, number of health facilities visited before definitive diagnosis, and case-finding method were related to LHSD (*p* < 0.05) (Table 3). However, after adjusting possible confounders, multivariate analysis confirmed that LHSD was more common in patients with cough and expectoration solely (OR: 1.482, 95%CI: 1.015–2.162) and those visiting ≥2 health facilities before diagnosis (2 health facilities: OR = 2.469, 95%CI: 1.239–4.920; ≥3 health facilities: OR = 8.306, 95%CI:4.032–17.111). Additionally, negative bacteriological results were independently associated with a higher risk of LHSD (OR = 1.485, 95%CI: 1.060–2.080) (Table 4). Subgroup analysis revealed no statistically significant interaction effects between clinical symptoms, the number of health facilities visited prior to diagnosis, and bacteriological results in their association with LHSD (all interaction *p*-values >0.05).

**Table 3:** Univariate analysis for health system delay in pulmonary tuberculosis diagnosis (*N* = 958).

Variable	No. of cases	No. of LHSD	Percent	*χ* ^2^	*p*-value
Sex				0.180	0.671
Male	657	134	20.4		
Female	301	65	21.6		
Age (year)				5.654	0.059
0–34	262	44	16.8		
35–64	499	104	20.8		
65–	197	51	25.9		
Education level				2.559	0.110
Junior middle school or below	633	141	22.3		
Above junior middle school	325	58	17.8		
Marital status				6.045	0.049
Unmarried	170	26	15.3		
Married or cohabitate	707	150	21.2		
Divorce or widow	81	23	28.4		
Occupation				11.954	0.063
Agricultural workers	324	78	24.1		
Industrial workers	168	37	22.0		
Cadre and staff^a^	69	8	11.6		
Students	60	5	8.3		
Retiree	58	12	20.7		
Houseworkers and un-employed	174	39	22.4		
Others	105	20	19.0		
Residence				3.737	0.053
Urban	563	105	18.7		
Rural	395	94	23.8		
Symptoms				12.702	0.002
Others^b^	509	106	20.8		
Only cough and expectoration	250	67	26.8		
Asymptomatic	199	26	13.1		
TB treatment history				1.428	0.232
Yes	72	11	15.3		
No	886	188	21.2		
Number of facilities visited before definitive diagnosis				64.097	<0.001
1	118	10	8.5		
2	647	110	17.0		
≥3	193	79	40.9		
Bacteriological results				3.271	0.070
Positive	459	84	18.3		
Negative	499	115	23.0		
Case-finding				7.236	0.007
Active	190	26	13.7		
Passive	768	173	22.5		
Diagnosis period				0.711	0.399
Before the epidemic	478	94	19.7		
During the epidemic	480	105	21.9		

LHSD, long health system delay. ^a^Including teachers, medical staff, cadres and other brain-based laborers. ^b^Patients with PTB symptoms except the ones with cough and expectoration only.

**Table 4:** Multivariate analysis for health system delay in pulmonary tuberculosis diagnosis (*N* = 958).

Variable	*β*	S.E.	Wald *χ*^2^	*P*	OR (95%CI)
Age (year)					
0–34					Reference
35–64	−0.123	0.271	0.208	0.649	0.884(0.520–1.502)
65–	−0.005	0.331	0.000	0.989	0.995(0.520–1.905)
Education level					
Junior middle school or below					Reference
Above junior middle school	0.183	0.228	0.644	0.422	1.201(0.768–1.878)
Marital status					
Unmarried					Reference
Married or cohabitate	0.206	0.309	0.445	0.505	1.229(0.671–2.249)
Divorce or widow	0.514	0.41	1.575	0.209	1.673(0.749–3.734)
Occupation					
Agricultural workers					Reference
Industrial workers	0.058	0.258	0.050	0.823	1.059(0.639–1.757)
Cadre and staff^a^	−0.875	0.463	3.566	0.059	0.417(0.168–1.034)
Students	−1.070	0.578	3.427	0.064	0.343(0.110–1.065)
Retiree	−0.484	0.408	1.408	0.235	0.616(0.277–1.371)
Houseworkers and un-employed	−0.103	0.254	0.166	0.684	0.902(0.548–1.483)
Others	−0.157	0.327	0.231	0.631	0.855(0.451–1.621)
Residence					
Urban					Reference
Rural	0.066	0.194	0.115	0.735	1.068(0.730–1.561)
Symptoms					
Others^b^					Reference
Only cough and expectoration	0.393	0.193	4.158	0.041	1.482(1.015–2.162)
Asymptomatic	−0.237	0.343	0.477	0.490	0.789(0.403–1.545)
Number of facilities visited before definitive diagnosis					
1					Reference
2	0.904	0.352	6.603	0.010	2.469(1.239–4.920)
≥3	2.117	0.369	32.962	<0.001	8.306(4.032–17.111)
Bacteriological results					
Positive					Reference
Negative	0.395	0.172	5.274	0.022	1.485(1.060–2.080)
Case-finding					
Active					Reference
Passive	0.258	0.34	0.573	0.449	1.294(0.6640–2.520)

OR, odds ratio; CI, confidential interval. ^a^Including teachers, medical staff, cadres and other brain-based laborer. ^b^Patients with PTB symptoms except the ones with cough and expectoration only.

## 4 Discussion

This study represents an updated nationwide multi-center survey on the HSD of PTB diagnosis, the results indicate that the HSD of PTB diagnosis is at a relatively low level in China, and no impact of the COVID-19 epidemic on HSD was observed. It is still noteworthy that patients presenting non-specific cough and expectoration, visiting multiple facilities before definitive diagnosis, and with negative bacteriological results were identified as risk factors for LHSD.

In our study, the HSD was 4 (1–10) days. This was consistent with a systematic review published in 2021, the HSD in five upper-middle-income countries with a high TB burden was 4 (95% confidence interval: 2–4) days (27). However, a multi-center study based on 20 hospitals in China found that the HSD was 20 (7–72) days (28), which is much longer than our survey result. The most likely reason for this large gap is that the participants in the former survey were diagnosed by TB-designated hospitals at the prefecture or provincial level, whereas the majority of patients with PTB in China are diagnosed and cured by TB-designated hospitals at the county level. Patients with complex conditions that are difficult to diagnose are referred or self-directed to higher-level TB hospitals (29), which lasts more time until diagnosis. Our survey results were also lower than those of a survey conducted in two districts of Beijing in 2021, which showed the HSD was 8 (0–18) days (30). The likely cause is Beijing’s abundant health resources attracting a high proportion of out-of-town TB patients (31), and they may have consulted locally health facilities but faced prolonged un-diagnosis periods, leading to longer HSD. The relatively low HSD in China can be attributed to three reasons. First, China’s TB prevention and control system with clear responsibilities and efficient coordination. Detailed regulations have been established for the reporting and referral of suspected PTB discovered in non-TB-designated hospitals, and corresponding tracking measures are in place for those not properly referred, shortening the time from the patient’s initial visit to definitive diagnosis (29). Second, improved self-health awareness and economic status promote the suspects promptly visit TB-designated hospitals for further diagnosis and treatment after detecting abnormalities during the initial visit (32). Third, according to “13th Five-Year Plan” for TB Control (31), at least 70% of county-level TB-designated hospitals were required to possess molecular biological diagnostic capabilities. This technique (e.g., GeneXpert), characterized by high sensitivity and rapid detection speed (33), has facilitated early diagnosis for PTB patients.

It is worth mentioning that, 13 patients with fever symptoms underwent COVID-19 quarantine measures without experiencing LHSD. There may be a detection symptom bias here, which potentially overstated the role of COVID-19 quarantine measures in shortening the HSD, as even without the COVID-19 quarantine measures, these PTB patients could still had been diagnosed earlier. However, the role of these stringent quarantine measures in promptly identifying some patients with fever symptoms is undeniable. Nevertheless, there is also a risk that patients with fever might be overlooked after testing negative for SARS-CoV-2 nucleic acid tests, leading to a prolongation of their diagnostic time. This highlights the importance of concurrent screening for PTB during future epidemic of similar infectious diseases (34). Over all, we observed no statistical difference in HSD for patients with PTB diagnosed before and during the COVID-19 epidemic, which according to a survey in Ningxia (20). The most plausible explanation is that NPIs and patients fearing of SARS-CoV-2 infection can lead to delays in seeking medical (35, 36), but once patients were able to access the health system, these impact on diagnostic delays is relatively minor.

The current study also showed that sex, age and other socio-demographic factors were not associated with the LHSD, which is consistent with the findings of a cross-sectional survey conducted in the Gurage and Siltie regions of southern Ethiopia (37). Similarly, interview-based studies conducted in Tanzania and Italy also revealed that there was no correlation between the patients’ demographic and sociological characteristics and HSD (38, 39). This consistent evidence across diverse settings suggests that HSD in PTB is primarily influenced by health provider’ factors rather than patient characteristics. Specifically, the timeliness of PTB diagnosis appears to depend largely on the diagnostic capacity of the health facilities patients visited.

We found that compared to patients with PTB presenting solely with cough and expectoration, those exhibiting other PTB symptoms were diagnosed more promptly, consistent with previous studies (40, 41). If patients have more specific symptoms of PTB, such as chest pain and hemoptysis, it would not only attract patients’ attention and shorten the time to seek healthcare but may necessitate health providers to investigate PTB early. Cough plays a critical role in the MTB transmission (42), warranting careful attention. However, the symptoms of sole cough and expectoration caused by PTB are often clinically similar to those of other respiratory diseases, posing challenges for differentiation (43). This study found that more than a quarter of these PTB patients experienced LHSD. When combined with the patient delay, it can be inferred that many patients experienced cough and expectoration for more than 2 weeks. Therefore, targeted training programs are imperative to enhance the awareness of health providers regarding patients with chronic respiratory symptoms, ensuring that PTB-related examinations are conducted when necessary to facilitate early detection of PTB cases.

Our analysis revealed a strong association between more health facility visits and LHSD, with patients visiting two or more facilities demonstrating significantly higher HSD incidence. This finding aligns with the reports from Ghana (44), Iran (45) and Afghanistan (46), where multiple visits to health care providers strongly predict increased diagnostic delay. Notably, similar to a domestic study, we observed a dose–response relationship, wherein the likelihood of LHSD increased proportionally with the number of facilities visited (15). The existing policy stipulates that tuberculosis patients should be treated centrally at TB-designated hospitals. Suspected cases from other health facilities must be referred to TB-designated hospitals for confirmation, registration, and treatment (47). Compared with other health facilities, TB-designated hospitals are more professional in PTB diagnosis, and require a shorter time for diagnosis (48). Our findings support this, showing that over 95% of patients initially visiting TB-designated hospitals received definitive diagnoses there. Thus, initial visits to TB-designated hospitals or prompt referral from other facilities could significantly reduce HSD. However, patient pathway analysis revealed that only 12.3% of patients chose a TB-designated hospital for their first visit, this value is close to a systematic review that encompassed 9,891 PTB patients in China (13%) (23). The ability of non-TB-designated facilities to identify and refer suspected PTB cases is critical. Yet, among patients initially visiting other facilities, particularly grassroots-level ones, only 50% selected TB-designated hospitals as their secondary facility. This indicates that primary healthcare providers failed to identify half of these patients, leading to further visits to non-TB-designated hospitals, additional referrals, and prolonged HSD. These findings highlight significant deficiencies in presumptive PTB recognition at grassroots facilities (49, 50), and necessitates the targeted training for grassroots doctors to enhance their ability to identify PTB.

In our study, patients with negative bacteriological results demonstrated a significantly higher likelihood of experiencing LHSD. This finding is similar to a study conducted in the Taiwan area (41). The association between negative bacteriological results and LHSD can be attributed to several factors. Primarily, the bacteriological detection of MTB serves as a critical component for the timely and accurate diagnosis of PTB (51). Furthermore, current clinical guidelines recommend a 14-day course of non-specific broad-spectrum antibiotic therapy for suspected PTB cases with negative bacteriological results prior to definitive diagnosis (29). This diagnostic approach, while clinically justified, potentially extends the time to diagnosis and may contribute to LHSD. The findings underscore the critical need to implement bacteriological diagnostic technologies for PTB to effectively mitigate HSD.

## 5 Strength and limitation

This nationwide survey provides valuable insights into HSD in the diagnosis of PTB patients in China. However, several limitations should be acknowledged. First, despite our implementation of consecutive enrollment based on registration numbers, a small proportion of patients were excluded due to loss to follow-up, mortality, refusal to participate, or incomplete data. Although comparative analysis revealed no significant differences in age distribution or gender composition between analyzed and non-analyzed participants, the potential for selection bias cannot be entirely ruled out. Second, our study was limited to patients notified at county-level TB-designated hospitals, excluding those notified at municipal or higher-level TB-designated hospitals. This exclusion criterion may introduce a degree of selection bias, potentially limiting the generalizability of our findings to the broader PTB patient population. Third, while we employed medical record verification to supplement and validate patient recall during the questionnaire administration, the retrospective nature of our study design inevitably introduces some degree of recall bias regarding illness progression and patient pathways. These limitations notwithstanding, our study provides crucial epidemiological data that contribute to the understanding of HSD in PTB management within the Chinese health system.

## 6 Conclusion

Health system delay in PTB diagnosis remains at a relatively low level in China. No significant impact on the HSD from the COVID-19 epidemic has been noted, although a small number of febrile patients with PTB were diagnosed in timely manner through COVID-19 quarantine protocols. The study identified patients with only cough and expectoration, contacted ≥2 health facilities, and negative bacteriological results increased the risk of LHSD. Based on these findings, we recommend the following interventions to further reduce HSD: Implementing targeted training programs to enhance health providers’ awareness of chronic respiratory symptoms and maintain vigilance for PTB; strengthening the presumptive PTB patients recognition capabilities of grassroots health facilities, and promoting the use of MTB bacteriological technologies. These evidence-based strategies could significantly improve early PTB detection and reduce HSD, ultimately contributing to better TB control outcomes in China.

## Data Availability

All data relevant to the study are included in the article and can be obtained from the corresponding authors upon reasonable request.
